# Comparison of Holmium:YAG and Thulium Fiber Lasers on the Risk of Laser Fiber Fracture

**DOI:** 10.3390/jcm10132960

**Published:** 2021-06-30

**Authors:** Audrey Uzan, Paul Chiron, Frédéric Panthier, Mattieu Haddad, Laurent Berthe, Olivier Traxer, Steeve Doizi

**Affiliations:** 1 Sorbonne Université, GRC n°20, Groupe de Recherche Clinique sur la Lithiase Urinaire, Hôpital Tenon, F-75020 Paris, France; audrey.uzan@aphp.fr (A.U.); p.chiron@laposte.net (P.C.); frederic.panthier@aphp.fr (F.P.); mattieu.haddad@gmail.com (M.H.); olivier.traxer@aphp.fr (O.T.); 2 Sorbonne Université, Service d’Urologie, AP-HP, Hôpital Tenon, F-75020 Paris, France; 3 PIMM, UMR 8006 CNRS-Arts et Métiers ParisTech, 151 bd de l’Hôpital, F-75013 Paris, France; laurent.berthe@ensam.eu

**Keywords:** Ho:YAG laser, thulium fiber laser, laser fiber, lithotripsy, urolithiasis, ureteroscopy

## Abstract

Objectives: To compare the risk of laser fiber fracture between Ho:YAG laser and Thulium Fiber Laser (TFL) with different laser fiber diameters, laser settings, and fiber bending radii. METHODS: Lengths of 200, 272, and 365 μm single use fibers were used with a 30 W Ho:YAG laser and a 50 W Super Pulsed TFL. Laser fibers of 150 µm length were also tested with the TFL only. Five different increasingly smaller bend radii were tested: 1, 0.9, 0.75, 0.6, and 0.45 cm. A total of 13 different laser settings were tested for the Ho:YAG laser: six fragmentation settings with a short pulse duration, and seven dusting settings with a long pulse duration. A total of 33 different laser settings were tested for the TFL. Three laser settings were common two both lasers: 0.5 J × 12 Hz, 0.8 J × 8 Hz, 2 J × 3 Hz. The laser was activated for 5 min or until fiber fracture. Each measurement was performed ten times. Results: While fiber failures occurred with all fiber diameters with Ho:YAG laser, none were reported with TFL. Identified risk factors of fiber fracture with the Ho:YAG laser were short pulse and high energy for the 365 µm fibers (*p* = 0.041), but not for the 200 and 272 µm fibers (*p* = 1 and *p* = 0.43, respectively). High frequency was not a risk factor of fiber fracture. Fiber diameter also seemed to be a risk factor of fracture. The 200 µm fibers broke more frequently than the 272 and 365 µm ones (*p* = 0.039). There was a trend for a higher number of fractures with the 365 µm fibers compared to the 272 µm ones, these occurring at a larger bend radius, but this difference was not significant. Conclusion: TFL appears to be a safer laser regarding the risk of fiber fracture than Ho:YAG when used with fibers in a deflected position.

## 1. Introduction

Since its introduction in the 1990s, Ho:YAG laser has become the reference point for lasers for lithotripsy in urology because of its property to fragment all stone compositions, efficiencies and safety profiles [1,2,3]. Recently, a new laser has been released: the Super Pulsed Thulium Fiber Laser (TFL), with potential advantages over Ho:YAG laser such as higher ablation volumes during lithotripsy and production of thinner particles [4,5,6,7,8]. These two lasers use low hydroxyl silica optical fibers to transmit the laser beam to the stone [4,5,9,10]. During laser lithotripsy with flexible ureteroscopy (f-URS), laser fiber rupture may occur especially for lower pole stones treatment, resulting in working channel perforation and subsequent endoscope repair. Some studies reported risk factors of laser fiber fracture with Ho:YAG laser while bending: the diameter of the bend and high pulse energy [11,12]. While Ho:YAG laser and TFL are currently used for lithotripsy during f-URS, there is a lack of comparative study regarding the risk of laser fiber fracture during laser activation in a deflected position. Thus, we aimed to compare the risk of laser fiber fracture between Ho:YAG laser and TFL with different laser fiber diameters, laser settings, and fiber bending radii.

## 2. Materials and Methods

### 2.1. Laser Fibers

Single use laser fibers of a unique manufacturer (Rocamed, Monaco) with core diameters of 200, 272, and 365 μm were used for both laser systems to avoid any confusion due to a variability in laser fibers characteristics. Additionally, 150 µm laser fibers were also tested with the TFL only.

### 2.2. Laser Systems

A 50 W Super Pulsed TFL generator (IPG Photonics, Fryazino, Russia) with a wavelength of 1940 nm was compared to a 30 W Ho:YAG laser (MH01-ROCA FTS-30W, Rocamed, Monaco) with a wavelength of 2120 nm. A total of 13 different laser settings were tested for the Ho:YAG laser: 6 fragmentation settings with a short pulse duration, and 7 dusting settings with a long pulse duration. A total of 33 different laser settings were tested for the TFL. Since TFL offers lower energies and higher frequencies than current Ho:YAG lasers, we aimed to evaluate these specificities. Three laser settings were common to both lasers: 0.5 J × 12 Hz, 0.8 J × 8 Hz, 2 J × 3 Hz. All laser settings tested are presented in Table 1.

### 2.3. Experimental Setup

**Figure 1:**
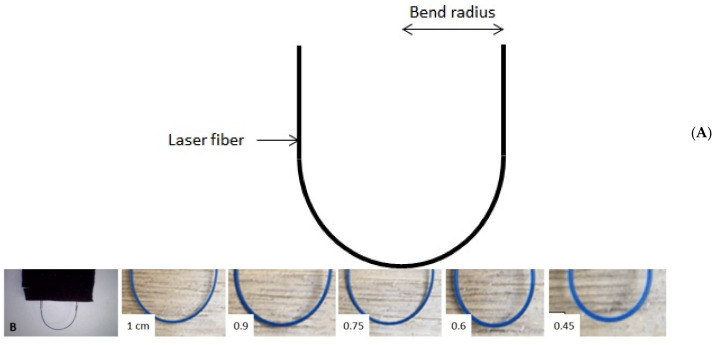
(**A**) Fiber bending radius, (**B**) Fiber bending radii tested.

The laser fibers were supported by soft silicone tubes, secured by plastic screws (to hold the fibers without causing damage). Failure threshold testing was done by bending fibers to 180° with an initial radius of 1 cm, Figure 1A,B. In total, five different increasingly smaller bend radii were tested: 1, 0.9, 0.75, 0.6, and 0.45 cm. The choice of the minimal bending radius (0.45 cm) was based on the fact that we measured the most acute angle over several cases that a flexible ureteroscope might deflect for lower pole lithotripsy in difficult anatomical situations. Subsequent radii were randomly chosen to test wider values mimicking calices easier to navigate through. The laser was activated continuously for 5 min or until fiber fracture. Each measurement was performed ten times.

### 2.4. Statistical Analyses

The Mann–Whitney test was used for comparisons between groups. All tests were conducted using the R Software, version 4.0.3. A *p*-value of 0.05 or less was considered significant.

## 3. Results

We did not report mechanical failure by bending the fibers alone. All fractures occurred after laser energy application.

### 3.1. Ho:YAG Laser

#### 3.1.1. Dusting Settings

**Figure 2:**
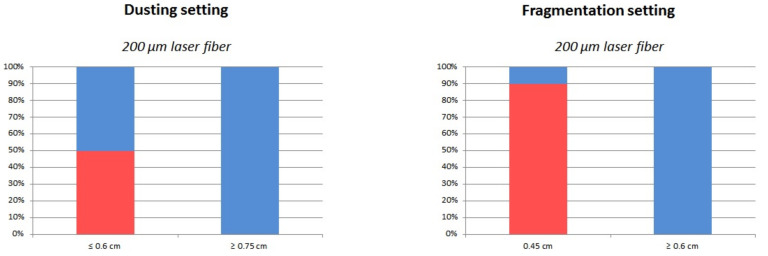

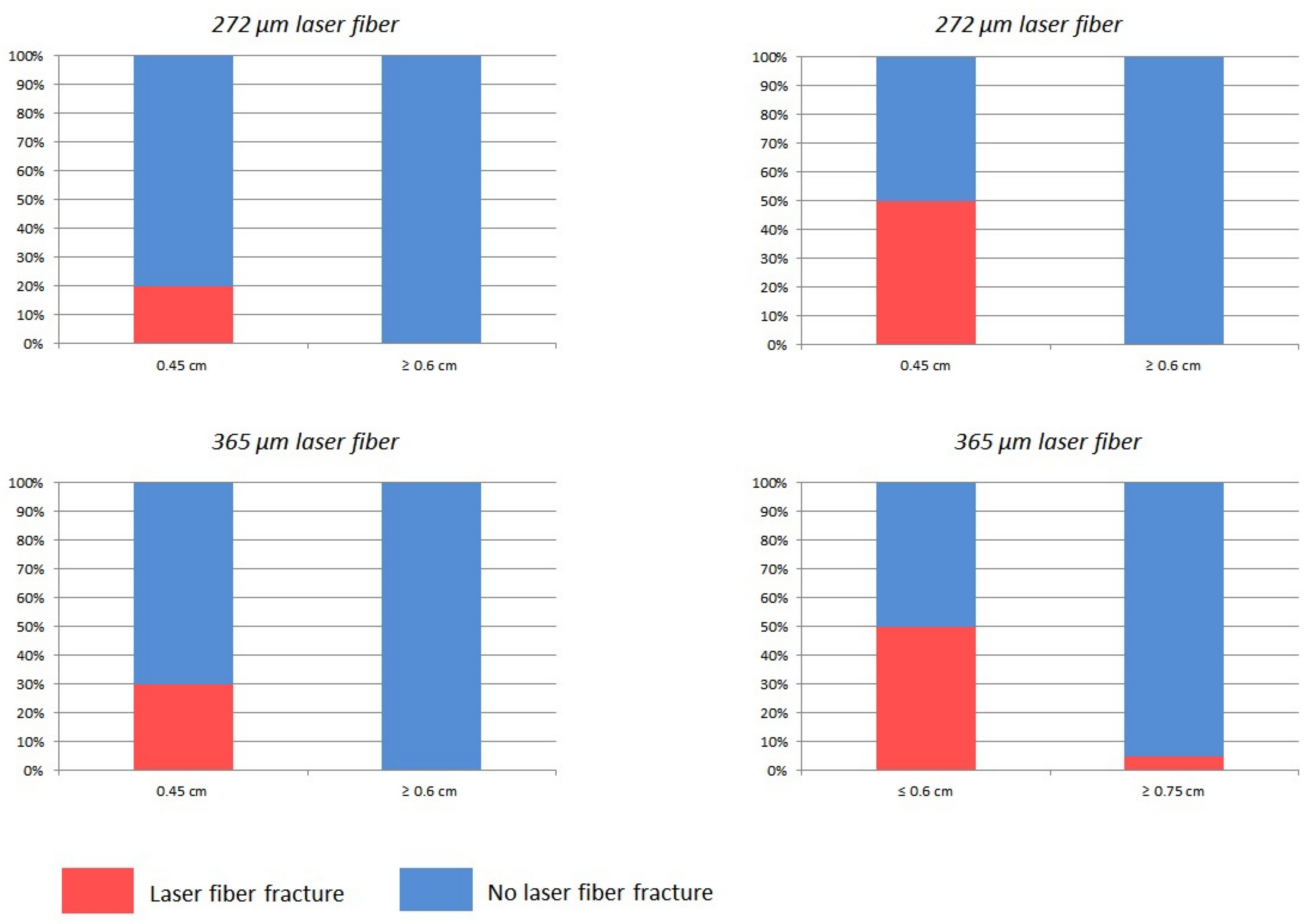
Proportion of fiber failures with Ho:YAG laser according to laser setting, fiber diameter, bending radius.

For the 200 µm fibers, the fracture rate was 50% at bending radius ≤0.6 cm, while none broke at radius ≥0.75 cm. For the 272 and 365 µm fiber diameters, fractures occurred only with a bending radius of 0.45 cm. A total of 20% of the 272 µm and 30% of the 365 µm fibers broke at a bend radius of 0.45 cm, Figure 2.

#### 3.1.2. Fragmentation Settings

Of the 200 and 272 µm fibers, there was no fracture for a bend radius ≥0.6 cm. While 90% of the 200 µm fibers broke at a radius of 0.45 cm, 50% of the 272 µm did. The 365 µm fibers broke more frequently at ≤0.75 cm. A total of 5% and 50% of 365 µm laser fibers broke with a bending radius of ≥0.75 and ≤0.6 cm, respectively, Figure 2.

#### 3.1.3. Identification of Risk Factors of Fiber Failure

Short pulse and high energy were significant risk factors of fiber fracture for the 365 µm fibers (*p* = 0.041), but not for the 200 and 272 µm fibers (*p* = 1 and *p* = 0.43, respectively). High frequency was not a risk factor of fiber fracture for all fiber core diameters.

Fiber diameter also seemed to be a risk factor of fracture. The 200 µm fibers broke more frequently than the 272 and 365 µm ones (*p* = 0.039). There was a trend for a higher number of fractures with the 365 µm fibers compared to the 272 µm ones, these occurring at a larger bend radius, but this difference was not significant.

### 3.2. TFL

Irrespective of the laser fiber diameter, laser settings, and bending radius, no fiber fracture occurred with the TFL.

### 3.3. Ho:YAG versus TFL

Irrespective of the laser settings, the fiber diameter and the bend radius, there was a significant risk of fiber fracture with the Ho:YAG laser compared to the TFL.

## 4. Discussion

The current study demonstrated a significant risk of fiber fracture with the Ho:YAG laser compared to the TFL in a deflected position. This result is of importance because nowadays f-URS has become a modality of choice for the treatment of kidney stones [13]. While Ho:YAG laser is currently the gold standard for lithotripsy during f-URS, TFL appears as an efficient alternative [14]. For both lasers, the laser energy is delivered to the target through a low hydroxyl silica fiber [9]. This laser fiber consists of a silica core through which the laser energy is transmitted. This core is surrounded by a layer called cladding that is essential for the efficient delivery of laser energy. This cladding is made of similar material to the core but has a different refractive index. Thus, the laser beam is reflected at the cladding–core interface. This process is called total internal reflection [9,10]. The most external part of the fiber is called jacket and encases the core and cladding. Its function is to protect the glass components of the fiber. When the fiber is bent, such as in lower pole stone treatment during f-URS, a small amount energy may leave the core to the cladding, and subsequently leak into the jacket. This condition represents a loss of total internal reflection of the laser energy, and once energy leaks into the jacket, fiber failure can occur due to thermal breakdown [15,16,17]. Prior studies demonstrated that the fibers do not fail with mechanical stress alone but rather fail when the laser is activated with the fiber in a deflected position. Consequences of such fiber failures are working channel perforations during laser activation, which represents an important cause of f-URS damage [18]. Several studies focused on the risk factors of fiber fracture in a deflected position with Ho:YAG laser [11,12,19,20,21,22,23]. They reported contradictory results regarding the influence of fiber diameter, bend radius, laser settings, and even for a same type of fiber from a specific manufacturer [12,20,21,22]. For example, while some authors reported that medium core fibers were prone to higher rates of failure than small core fibers, other studies did not document a correlation between increasing fiber diameter and fracture [11,20]. However, all the studies found that the resistance to fracture varies greatly among fiber manufacturers [12,20,21,22].

Similarly to Mues et al., we did not report mechanical failure by bending the fibers alone [21]. This means that failure is the consequence of loss of total internal reflection during laser activation in a bent fiber.

### 4.1. Ho:YAG Laser

The current study found that small core fibers (200 µm) were prone to a higher rate of fracture and failed at a larger bend radius (≤0.6 cm) than 272 and 365 µm fibers in dusting setting (0.45 cm only). Surprisingly, no 200 µm fiber failure occurred at a bend radius ≥0.6 cm in fragmentation setting, but there was a higher proportion of fractures than in dusting setting (90% versus 50%, respectively). Thus, we found that small core fibers failed significantly more often than the 272 and 365 µm ones. These results are consistent with the report by Mues et al., and may be explained by the beam profile of the Ho:YAG laser [21]. Indeed, the Ho:YAG laser beam does not couple small core fibers (<200 µm), and the risk may be overfilling the fiber core and leak laser energy to the fiber cladding, which can damage the fiber [4,5,24,25]. Thus, the use of small core fibers require the funneling of laser beam. As consequence, Ho:YAG laser is typically limited to larger fiber diameters (270–500 μm).

For the 272 and 365 µm fibers, we found similar results than Haddad et al., the 272 µm fibers failed at a smaller diameter than the 365 µm in fragmentation setting, but not in dusting setting.

Although 200 µm fibers are more flexible and may be more suitable for the treatment of lower pole stones during f-URS, they are more prone to failure when lasering. Thus, 272 µm core fibers seem a safer option for lower pole f-URS with Ho:YAG laser.

Finally, similarly to Knudsen et al., we found that the tightness of the fiber bend radius increases the risk of fiber failure as well as pulse energy for the 365 µm only [12]. This means that for a fixed bending radius, if the pulse energy increases, the amount of energy leaking the core to the cladding increases, and thus the risk of fiber fracture. On the contrary, Lusch et al. reported a trend for less fiber fracture at long pulse mode, high energy, low frequency in the small core fibers (200, 272/273 µm). Contrary to Vassar et al., we did not report an increase failure rate when the laser pulse energy increases with 272 µm fibers compared to the 365 µm [26].

### 4.2. TFL

Until now, no study has evaluated the risk of laser fiber fracture with the TFL. We found that, irrespective of the laser fiber diameter, laser settings, and bending radius, no fiber fracture occurred. These results may be explained by the beam profile and the peak power of the TFL. Contrary to the solid state Ho:YAG laser, the laser beam of the TFL originates within a small (18–25 μm) core of the thulium-doped silica optical fiber, which is about 100 times smaller in diameter than Ho:YAG laser. Furthermore, the TFL provides a near single mode Gaussian spatial beam profile, more uniform and symmetrical than the multimodal beam produced by the Ho:YAG laser [24]. Thus, even thinner laser fibers (150 µm) can be used with TFL. As consequence, total internal reflection may be respected in all fiber core diameters, with no leakage of energy through the cladding and jacket, which reduce the risk of fiber fracture. Moreover, peak power may also explain the absence of fracture with TFL. Indeed, the differences in fiber fracture rates between the two lasers systems may be explained by the constant higher peak power with the Ho:YAG laser compared to the TFL, regardless of the laser settings [27]. While peak power is directly correlated to the energy level with Ho:YAG laser and decreases with increased pulse duration, this remains constant with TFL. Furthermore, the pulse shape is also different with a flat and uniform shape for the TFL and a spike with an overshoot for the Ho:YAG laser [27]. Thus, the treatment of lower pole stone with TFL may be safer than with Ho:YAG laser, regardless of fiber diameter, bend radius, and laser settings.

Our study has several limitations, including the use of laser fibers from a unique manufacturer. However, by using exactly the same laser fiber manufacturer, it was possible to show the differences between both laser technologies, without risking the additional bias that using laser fibers from different origins might introduce. Yet, since great differences regarding size, flexibility, and resistance to fracture with bending among manufacturers exist, more optical fibers should be tested to ascertain our results with TFL. Although, laser fiber manufacturers provide short term minimum bending radius, we did not respect them in our tests since it is not possible to respect these minimal values in real conditions, especially in a difficult lower calyx access. Indeed, short term minimum bending radii were ≥13 mm, ≥17 mm, and ≥21 mm for the 200, 272, and 365 µm laser fibers tested, respectively. Another limitation was the absence of power transmission measurement. With transmission values, a quantitative correlation of core diameter, bending radius and losses might be possible. Lastly, laser activation duration was fixed at 5 min or until fiber fracture, which has resulted in different total energies delivered between powers tested. However, this might affect the results with Ho:YAG laser only, since no fiber fracture occurred with TFL.

## 5. Conclusions

The is the first study comparing the risk of fiber fracture with different laser fiber diameters, laser settings, and fiber bending radii between the Ho:YAG laser and TFL. While fiber failures occurred with all fiber diameters with Ho:YAG laser, none was reported with TFL. Further studies testing fibers from different manufacturers are needed to ascertain these results.

## Figures and Tables

**Table 1:** (**A**): TFL laser settings; (**B**): Ho:YAG laser settings.

**A. TFL Settings**			
	**6 W**	**25 W**	**50 W**
*Fine dusting (peak power = 125 W)*			
0.025 J	240 Hz	1000 Hz	2000 Hz
0.05 J	120 Hz	500 Hz	1000 Hz
0.1 J	60 Hz	250 Hz	500 Hz
0.15 J	40 Hz	167 Hz	333 Hz
*Dusting (peak power = 125 W)*			
0.2 J	30 Hz	125 Hz	250 Hz
0.5 J	12 Hz	50 Hz	100 Hz
0.8 J	7.5 Hz	31.3 Hz	62.5 Hz
*Fragmentation (peak power = 500 W)*			
1 J	6 Hz	25 Hz	50 Hz
2 J	3 Hz	12.5 Hz	25 Hz
4 J	1.5 Hz	6.3 Hz	12.5 Hz
6 J	1 Hz	4.2 Hz	8.3 Hz
**B. Ho:YAG Laser Settings**			
*Dusting (long pulse)*			
0.2 J	25 Hz		
0.5 J	3 Hz	12 Hz	15 Hz
0.8 J	3 Hz	8 Hz	15 Hz
*Fragmentation (short pulse)*			
1 J	3 Hz	5 Hz	15 Hz
2 J	3 Hz	8 Hz	12 Hz

## Data Availability

Data are available by contacting authors.
